# A Spicy Letter

**Published:** 1884-10

**Authors:** J. G. Hopkins

**Affiliations:** Atlanta, Ga.


### Correspondence.

---

### A SPICY LETTER IN WHICH A GEORGIA DOCTOR TELLS WHAT HE KNOWS ABOUT DR. WM. A. HAMMOND, OF NEW YORK, AND HIS TREATMENT OF A GEORGIA PATIENT.

DEAR JOURNAL: Please give me space through which to ventilate the fraud practiced upon patients sent from our State and the South generally to the erudite W. A. Hammond, of New York City.

It was my painful duty to take North recently a patient in whom I felt much interest and who, like myself, desired to "grasp at every straw" which presented the faintest shadow of hope. Sayre, Loomis, Otis, and others whom I would have preferred, were out of the city, so on account of Hammond's world-wide fame for the treatment of nervous diseases I sought him.

Having sent in my card and his readiness to receive me being announced, I entered his consultation room. The first object which fixed my gaze was the monarch himself, perched upon a large armed chair, the back of which, stretching far above his hoary head, bore the image of an Egyptian female and many unmistakable evidences of antique art. The walls and ceiling of the apartment were elaborately frescoed in a style which reigned supreme in the days of Herculaneum and Pompeii. Over 3,000 volumes bedecked the space between doors and windows, and in every nook and corner could be seen the work of the skilled sculptor, such as woman-headed horses and many other objects of the mythologic age. In the distance is heard a clock which strikes hours and half hours, and precedes each striking with sweet, enchanting music, which seems as though announcing the approach of the guardian angels of this superhuman neurologist. A gorgeous polycolored chandelier o'erhung the table and desk in the center of the room at which Lord Hammond communes with his prey. Upon this table there are a variety of images and things too numerous to mention, as well as a number of books which, lying open, stare the visitor in the face and smilingly point up to the Doctor and call him father. On his left is a pile of $20 bills, held down by an ill-shapen stone. Just across this *exchequer for the public gaze* is the chair in which all visitors are peremptorily told to "sit down."

As I entered, I said: This is Dr. Hammond, I suppose ?

"Yes sir."

Hopkins is my name, sir, and before I could complete my sentence, without the movement of another muscle, I was pointed to the seat at the treasury and told to "sit down."

I did so, and explained the object of my visit and the nature of the case. The young man was called and as he entered I said this is Mr. Blank, Dr. Hammond. Without another word or a movement *he too* was pointed to the $20 chair, which I had just vacated, and told to "sit down." I was shown one of the two chairs then unoccupied.

After a moment's pause a voice came down from that great high chair, saying,

"Well, what is the matter with you ?"

"I don't know, doctor; that is what I have come to you for."

"What are your symptoms? How do you complain?"

A short conversation ensued tending to an investigation of the case and the boy's hand was seized and the ball of the thumb punctured by some unseen instrument. A drop of blood was taken on a large glass cube and it, with the Doctor, disappeared. A few moments more and he returned, having had about time enough to throw the blood out of the window. "You have too much blood on your brain, sir," he said, "but I can cure you in a very short time. Come in here."

We now followed him into that mysterious chamber in which his wonderful cures are effected. The greater part of this room is occupied by his ponderous electrical machinery. He examined one eye with a large, double convex lens and a circular perforated reflector. and said: "Ah; there it is. Just as I said; congestion of the brain, I will relieve you in a few minutes. Here is a little instrument which, when put to your head, will locate the trouble. When applied, if this little needle you see here runs toward you there is too much blood in the back of your brain; if it runs from you, there is too much in the front." (This little instrument, as is known, only locates increased temperature of skin.) The little needle ran quickly towards the boy to thirty-five, this number being almost the maximum limit.

"Just as I told you; congestion of the base of the brain. Now, sir, sit up here."

Taking another stool, my patient asked: "What are you going to do now?" Standing behind him, the doctor said: "I am going to examine you," and in that self-same moment he dabbed to the nape of the boy's neck a large platinum point heated to a white heat, which, from the readiness of application, seemed to be always prepared. It is useless to say that the boy left that mystical stool without invitation. He was then put upon an insulated stool and filled with electricity, electric sparks being drawn from different parts of his body. This being over, the *little instrument* was again tried and it scarcely ran to ten, showing almost perfect relief from a congestion of the base of the brain of nine months' standing in the fabulously short space of about five minutes' time. Is not this the most remarkable diagnosis and cure you ever heard of? It is too ridiculous for comment.

We were ordered to return each day, with the most positive assurance of recovery, and that by Sunday (this being Tuesday) he would go home perfectly well and happy. The young gentleman was here dismissed, and I said to the Doctor that according to his own writings in his work on Cerebral Hyperæmia the most prominent symptom (imperfect sleep) was wanting in this case, for the boy slept the sweet, unbroken sleep of a new-born babe. He replied that since the writing of that book he had gained valuable information. A further attempt was made to discuss the matter, but I soon found that it was a topic upon which he could not be further engaged. If there was anything in either diagnosis or treatment, or if his manipulations were for moral effect, why did he not converse with me upon the subject? Having asked his charge and being told it was twenty dollars for first visit and ten dollars for each subsequent one, I handed him twenty dollars and left in disgust.

I went to Philadelphia in search of Dr. Wier Mitchell. He was in Europe, and I returned to Dr. H., hoping good from the moral treatment alone, for the medical treatment met with at his hands was substantially the same I had had the boy on for some time. Day after day we went through nearly the same circus, except that the actual cautery was omitted and the *clown* was a little more courteous. Each day we left him ten dollars, but such small amounts were never elevated to the exalted position of those twenties; nothing but a twenty ever went there, and to my *certain knowledge*, from a careful investigation, the number of twenty dollar bills increased as the day advanced whether there were any new cases or not.

The patient got no better, though that remarkable little instrument showed steady improvement until the needle now pointed to 7½ in the opposite direction, and the Doctor insisted that he was cured. Finding that he could not make the young man acknowledge that he was better, but rather insisted that he was worse, he simply said:

"'Tis all damned nonsense, and I shall write your father to send you to the asylum. Do you suppose God is going to help you and you sit down on your tail and not help yourself?"

But for the positive assurance of recovery and my hope in the moral effect of our visits, I should never have seen him but the once.

Dr. Hammond knew when he first saw that lad what the trouble was and knew what the result of his treatment would be. His experience is too widespread; his knowledge of medicine is too great for him not to have known.

There is a famous case which was, I believe, sent from Georgia to this doctor, in which a chicken's breast bone figures very largely. I wish I had space to tell it as related to me by New York doctors. When his eye meets this article he will quickly recall that bone experience and the death of the subject in a lunatic asylum, as well as the FEE for the feigned operations, which he poured into his greedy coffer.

I have written you this letter not in that spirit which characterizes a spleen venter, but simply to apprise Southern physicians of the character of the sagacious Hammond, who was at one time fired out of the United States army.

To physicians comment is unnecessary.

J. G. HOPKINS, M.D.

Atlanta, Ga., September, 1884.

